# Prebiotic RNA Network Formation: A Taxonomy of Molecular Cooperation

**DOI:** 10.3390/life7040038

**Published:** 2017-10-16

**Authors:** Cole Mathis, Sanjay N. Ramprasad, Sara Imari Walker, Niles Lehman

**Affiliations:** 1 Beyond Center for Fundamental Concepts in Science, Arizona State University, Tempe, AZ 85287, USA; cole.mathis@asu.edu (C.M.); sara.i.walker@asu.edu (S.I.W.); 2 Department of Chemistry, Portland State University, P.O. Box 751, Portland, OR 97202, USA; rsanjay@pdx.edu; 3 School of Earth and Space Exploration, Arizona State University, Tempe, AZ 85287, USA

**Keywords:** cooperation, prebiotic chemistry, taxonomy, ribozymes, RNA, origin of life

## Abstract

Cooperation is essential for evolution of biological complexity. Recent work has shown game theoretic arguments, commonly used to model biological cooperation, can also illuminate the dynamics of chemical systems. Here we investigate the types of cooperation possible in a real RNA system based on the *Azoarcus* ribozyme, by constructing a taxonomy of possible cooperative groups. We construct a computational model of this system to investigate the features of the real system promoting cooperation. We find triplet interactions among genotypes are intrinsically biased towards cooperation due to the particular distribution of catalytic rate constants measured empirically in the real system. For other distributions cooperation is less favored. We discuss implications for understanding cooperation as a driver of complexification in the origin of life.

## 1. Introduction

Recently, there has been a renewed interest in the importance of cooperation and collective behavior for both the emergence and evolution of biological complexity [1,2] and, importantly, for the origins of life itself [3]. It is becoming increasingly clear that one can trace the biological roots of cooperation down to the chemical level [4]. Molecular behaviors can be described in terms of game theoretic analyses with kinetic fitness payoffs that have direct relevance to the evolutionary fate of molecular populations [5,6]. It has also been suggested that cooperative effects may be responsible for the long-term stability of the living state [7].

**Figure 1:**
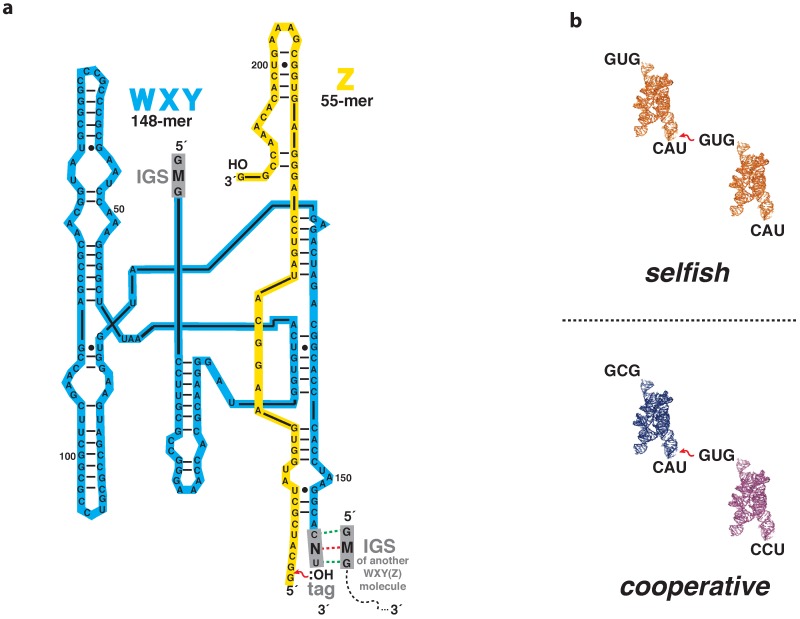
The *Azoarcus* ribozyme self assembly system. (**a**) The reaction between the 148-nt **WXY** RNA fragment and the 55-nt **Z** RNA fragment. This reaction is catalyzed by the binding of the internal guide sequence (IGS) of another catalyst (either a covalently-contiguous **WXYZ** molecule that had been previously assembled or a non-covalent **WXY-Z** complex) to the tag sequence on the 3′ end of the substrate **WXY** molecule. The key hydrogen bonding interactions are shown in green (invariant) and red (variable) dotted lines; (**b**) A comparison of selfish and cooperative assembly reactions by the use of two example pairs. In selfish assembly, the IGS (upper left of each molecule) of the substrate and the catalyst RNAs match; in cooperative assembly they do not match.

The *Azoracus* catalytic RNA covalent self-assembly system [8] allows us to explore these concepts directly both experimentally and computationally. In this system, cooperation arises due to the interaction among distinct genotypes leading to collective fitness gain. Briefly, in this system RNA molecules form an interaction network enabling the autocatalytic assembly of similar—and dissimilar—genotypes [9,10]. Genotype identity can be embodied in two three-nucleotide sequences at the 5′ and 3′ ends of the ca. 200-nucleotide ribozyme. The 5′ triplet is termed the internal guide sequence (IGS). IGS binding to a psuedo-complementary triplet at the 3′ end of another RNA determines the rate at which one ribozyme genotype assembles another from smaller fragments. A simplified version of this system in which only two fragments need to be covalently recombined to produce the ribozyme is depicted in Figure 1a. Here we describe the reaction as **WXY** + **Z→ WXYZ**, where **WXYZ** represents the full-length ribozyme. Cooperation, in this molecular context, is the event whereby one **WXY** genotype assembles another ribozyme with a distinct genotype; in other words, when the IGS sequences of the catalyst and its substrate do not match. Conversely, selfish assembly occurs when the IGS triplets of the catalyst and its substrate are the same [10] (Figure 1b). The balance between cooperative and selfish strategies is a key determinant in population evolution [11].

The origins of life likely required a kinetic competition among informational units prior to the advent of Darwinian evolution seen in contemporary life [12]. In a chemical soup this competition would have been played out in a network setting among a wide variety of molecular species. One approach to decomposing such network dynamics into smaller mechanistic elements is to use game theoretical methods to understand how pairs or triplets of genotypes interact in isolation. Game theory, in this context, is a mathematical framework in which the dynamical behavior of alternative reproductive strategies compete for limiting resources can be quantified through payoff matrices. The dynamics of such subnetworks can then be used to understand how larger chemical networks may behave. However, before this can be done, it is necessary to have a complete understanding of the rules that govern subnetwork growth and interaction.

**Figure 2:**
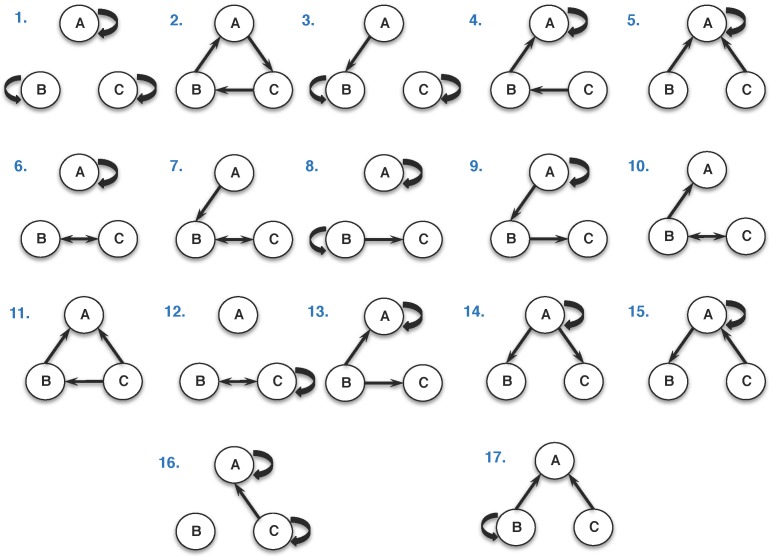
The 17 possible non-trivial 3-node, 3-edge network topologies. Topology 2 is the ”rock–paper–scissors” (RPS) scenario. **A**, **B**, and **C** denote distinct **WXY** RNA genotypes, while the arrows denote the ability of one genotype to covalently assemble another. For comparison, there are only four 2-node, 2-edge topologies, and we explored these in detail previously [6].

In the *Azoarcus* ribozyme system, evolvable networks can be composed of as few as three **WXY** genotypes. Previously we have explored the joint growth dynamics of a particular 3-membered network of such genotypes [6,9]. This network embodied the “rock–paper–scissors” (RPS) game theoretic dynamic, which has been also explored at a variety of biological levels [13,14]. In the RPS scenario, one genotype is superior to another by direct comparison, and so on around the cycle. However there are far more possible topologies than just RPS, depending on the directionalities and strengths of the various inter-genotype node interactions. In fact, there are 17 distinct 3-node, 3-edge topologies (including edges that are a self-loop), with RPS being but one of them (Figure 2).

The complexity of network dynamics is manifested by the constraints imposed on the processes that define the pairwise interactions of the members of the network itself. Understanding the emergence of these dynamics is of significant interest in prebiotic chemistry [12]. The topological artifacts of network graphs, e.g., edges and nodes, inform both the direction and magnitude of these interactions. Figure 2 represents a global overview of the classes of pairwise interactions possible in such three-member RNA networks. All possible triplet genotype combinations are represented by one of these 17 classes of global network topologies. In these networks, the constraints imposed are the real rate constants of self-assembly derived from previously demonstrated empirical data [6].

In this manuscript, we explore the hierarchical relationships among the types of molecular behavior (i.e., cooperative or selfish) in these three-genotype networks. Specifically, we are interested in the emergence of inter-genotype dynamics at the network level due to bond type and strength at the chemical level. Using empirically determined rate constants for the various IGS-substrate interactions, we computationally model the landscape of all possible genotypic interactions and explore RNA growth dynamics for each, producing a full picture of the dynamics of small networks that would be intractable to explore in the lab. By systematically modeling all 1, 2, and 3-genotype fitnesses within these simple topologies, and categorizing the various possible fitness benefits to individuals versus groups, we construct a taxonomy of cooperation that provides new insights into the molecular dynamics of cooperation that could have driven the emergence of life.

## 2. Results

### 2.1. Nomenclature and Chemistry

As in previous works [6,9,10,15], we designate each **WXY** RNA genotype by a two-letter name, MN. The first letter, *M*, denotes the middle nucleotide of the IGS, while the second letter, *N*, denotes the middle nucleotide of the target triplet on the 3′ end of the **WXY** fragment (Figure 1a). We term this latter triplet the “tag” sequence. The IGS–tag triplet–triplet binding through nucleotide pairing is the key chemical and informational interaction that determines the rate at which one genotype will catalyze the covalent assembly of another ribozyme [15]. The wildtype IGS sequence of the *Azoarcus* group I intron ribozyme is 5′-GUG-3′ [16], and the psuedocomplementary sequence to this in the tag would be 5′-CAU-3′. Although in principle all three positions could be varied, proper group I intron activity is highly dependent on a G•U wobble at the splice site [17,18], meaning that the first (5′) nucleotide of the IGS was best fixed as a guanosine, and the third (3′) nucleotide of the tag was best fixed as a uridine (Figure 1a). Moreover, we have found that variation at the middle nucleotide of the IGS–tag triplet is better tolerated than variation at either end [19]. Thus we focused only on variation in the middle position of the IGS–tag interactions (the red dotted line in Figure 1a) such that there are 16 possible **WXY** genotypes, i.e., 5′-${}_{GMG}$**WXY**${}_{CNU}$-3′. These can be abbreviated *MN*, such that, for example, the genotype 5′-${}_{G\underline{U}G}$**WXY**${}_{C\underline{A}U}$-3′ can be simply denoted UA.

In previous work, we measured all possible self-assembly rates for the 16 *MN* **WXY** genotypes when incubated with equimolar **Z** in 100 mM MgCl_2_ at 48 °C [6,15]. The rates are listed in Table 1 and serve as the empirical basis for all the estimated genotype–genotype edge strengths in the networks we examine herein. These rates vary over three orders of magnitude and exhibit a specific distribution of values, owing to the energetics of IGS–tag interactions. The goal of the current work is to test the causal chain of events from the H-bonding patterns that exist in the IGS–tag interaction to the patterns of cooperative or selfish behavior that manifest at the population level. To our knowledge this has never been explicitly done before. Specifically, we explored the differences in growth rates, as a measure of fitness, for genotypes when they are isolated, exist in pairs, or exist in triplets.

### 2.2. Classification and Taxonomy

There are 16 possible *Azoarcus* **WXY** genotypes, therefore there are 16Choose3 = 560 possible triplet groups, and within each of those 560 triplets there are 3Choose2 = 3 unique pairs. Each of these triplets can be classified according to the degree of cooperation exhibited by the triplet. One type of classification is simply to count the number of growth rates which were improved in the triplet relative to the individual rates. If all the genotypes in a triplet had a growth enhancement relative to their isolated growth rates, $R_{m}^{ijk}>R_{m}^{m}$ for m∈(i,j,k), we say that triplets has three cooperators and we call that triplet *cooperative*. If two genotypes improve their growth rates relative to the isolated condition, we say that triplet has two cooperators and we refer to that triplet as *semi-cooperative*. If only one growth rate improves relative to the isolated cases, it has one cooperator and we refer to that triplet as *selfish*, and if all the rates either remained constant or decreased in the triplet relative to the isolated rates, $R_{m}^{ijk}\leq R_{m}^{m}$ for m∈(i,j,k), it has 0 cooperators, and we call that triplet *antagonistic* (or in the case that all rates remain constant *neutral*). These classifications describe the degree to which all three genotypes gain a fitness advantage by being in a triplet as opposed to existing as isolated individuals. We refer to this as the first level of classification in our taxonomy.

There is another, finer, level of classification based on the number of growth rates which improved in the triplet relative to the pair-wise interactions. For any one of the 560 triplets, there are three possible pairs (ij), (ik), and (jk), the growth rate of each genotype in each pair (e.g., $R_{j}^{jk}$) can be compared to the growth rate of that triplet in the triplet ($R_{j}^{ijk}$), allowing for six comparisons per triplet. We can count the number of times a triplet growth rate is greater than a pair-wise growth rate for a given triplet, giving a number between 0 and 6. This number describes the degree to which pairs of genotypes gain a fitness advantage by being in a triplet as opposed to an isolated pair. For example, if a triplet scores a 0 on this level of classification, that means that the growth rates for all the genotypes in the triplet would have been higher in either of their two possible pairs, as compared to their growth rates in the triplet. While if a triplet scores a 6 on this level, every genotype has a higher growth rate in the triplet, as compared to any of its two possible pairs. Many different configurations can generate scores between 0 and 6; for example a score of 3 could be caused by one genotype in each possible pair having an increased rate in the triplet relative to those pairs, or by one pair having both members increase their growth rates in the triplet and another individual benefit from the triplet as compared to its possible pairs. We refer to this as the second level of classification.

**Figure 3:**
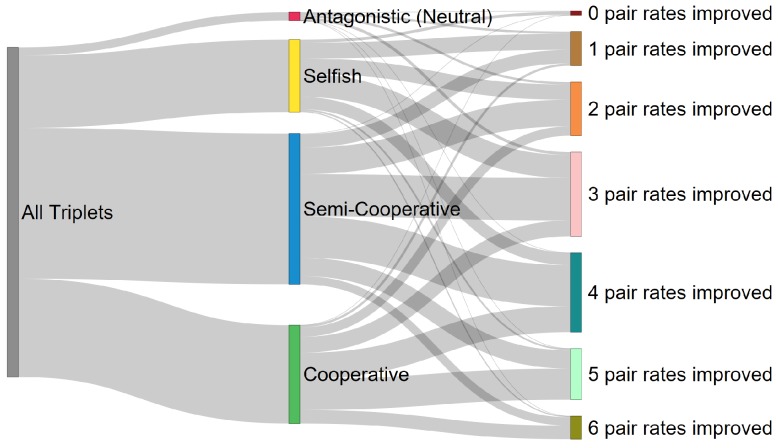
Taxonomy of molecular cooperation using the empirically derived rate constants. The left bar (gray) represents all possible 560 triplet genotype combinations. The bars in the center correspond to the first-level classifications, and the height of the bar indicates which fraction of all triplets fell into that category. The bars on the right show the second level classifications and the fraction of all triplets which fall into that category.

The overall “taxonomy” that results by considering these two levels of classification can be seen in Figure 3. One immediate result is that the taxonomy is skewed towards cooperation at both levels of analysis. There are more semi-cooperative and cooperative cases combined (256 + 167 = 423) than there are antagonistic and selfish (14 + 123 = 128). Likewise there are more cases where 4, 5, and 6 pairs are improved (261) compared to cases where 0, 1, or 2 pairs are improved (156) upon group inclusion. This bias is a combination of two factors. First, there are simply more combinatorial ways to be cooperative. This is due to the fact that none of the IGS–tag interactions are inhibitory in nature, so the presence of any other genotype in principle can contribute to an enhanced growth rate. In the finite resource simulations presented here, this effect is mitigated because the improvement gained by a small contribution to the catalytic rate is off-set by more rapidly decreasing resource abundances.

**Figure 4:**
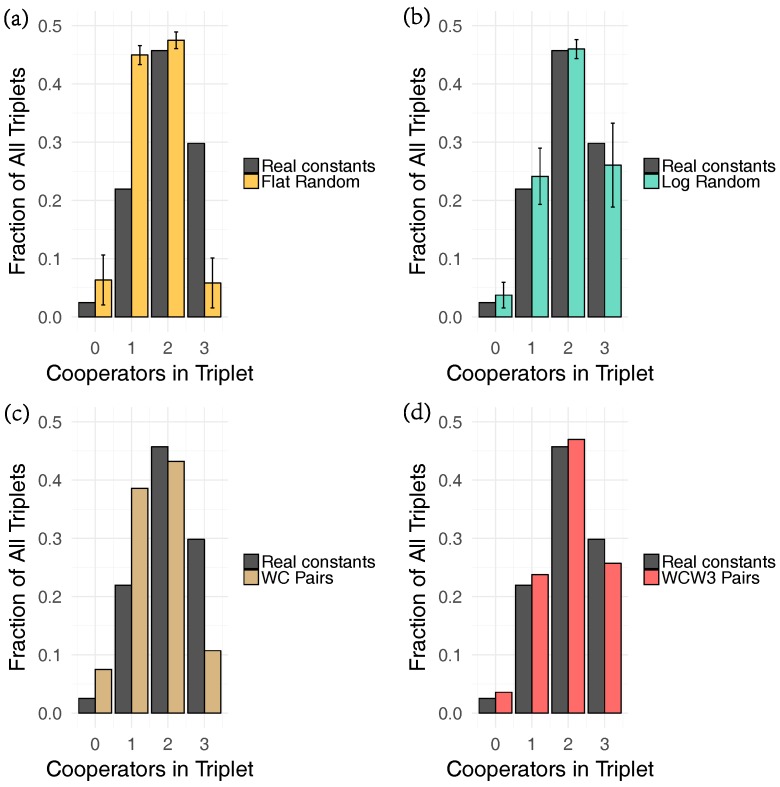
The effect of the distributions of ribozyme self-assembly rates on cooperation. (**a**) Flat random vs. real rate constants. The Empirically derived rate constants show an enhanced level of cooperative relative to a completely randomized control. (**b**) Log random vs. real rate constants. The empirically derived distribution of cooperation is well explained by a logarithmically distributed random distribution. (**c**) WC pairs vs. real. The empirically derived distribution of cooperation is not completely explained by including two values of rate constants. (**d**) WC with three wobble pairs vs. real. The empirically derived rate distribution of cooperation is well explained by including wobble pairs in a simplified set of rate constants.

Secondly and more critically, there is an influence based on the distribution of rates as seen in Table 1. We randomized the rate distributions in various ways to compare them against the empirical distribution of rates (Figure 4). In short, we tested a completely random (flat distribution), a heterogeneous distribution (random on a logarithmic scale), and a distribution which accounts for the discrepancy between Watson–Crick (WC) pairs and all other base pairing (WC distribution), as well as one which includes the effects of ‘wobble’ pairs (WCW3 distribution). For a complete description of the different distributions see Section 4. The degree of cooperation seen in the empirically derived system is much greater than would be expected if the catalytic rate constants were homogeneous (e.g., drawn from a random flat distribution). We also found that while the WC distribution was unable to produce the degree of cooperation seen in the empirically derived system, the WCW3 distribution agreed well with the empirically derived system. The heterogeneous distribution agreed well with the empirically derived system also, suggesting that the heterogeneity of the distribution, and not the particular order of the base pairs drives the observed cooperation. In particular we note that both the WCW3 and log random distributions yielded many more cooperative triplets relative to the WC and flat random distributions.

## 3. Discussion

By building a kinetic model of the *Azoracus* ribozyme **WXY** genotype system, we were able to investigate the distribution of cooperative effects in that system, as well as the drivers of that distribution. We found that the real system demonstrated an enhanced level of cooperation relative to a randomized control. We are also able to observe a diverse array of evolutionary trade-offs, as the number of unique genotypes in a system increases. We further found that these enhanced rates of cooperation were well-explained by the heterogeneous distribution of catalytic rate constants. These results have several consequences for understanding the evolution of cooperative catalytic networks.

While previous studies of collectively autocatalytic networks have provided topological constraints on such systems [12], results presented here provide new constraints on the dynamics of autocatalytic RNA networks. Prior investigations of autocatalytic chemical reaction networks find that they are rarely fixated in randomly assembled networks [20]. This rarity may be due (in part) to the uniform distribution of rate constants used in many of these studies. The results presented here suggest that the likelihood of observing such networks dynamically (either in the lab or in silico) would enhanced by using a heterogeneous distribution of rate constants.

By classifying the degree of cooperation using these two scales of organization (improvement from individual rates, and improvement from pair-wise rates), we can begin to understand how cooperation in the *Azoarcus* RNA system changes under aggregation (e.g., addition of more unique genotypes). We find that there are some genotype triplets which are fully cooperative, and score a 6 in the second level classification, meaning that all the genotypes grow faster in the triplet than they do alone, and all possible pairs of genotypes grow faster in the triplet than they do in pairs. Triplets of this kind would be expected to have an enhanced stability in the face of external perturbations [21]. There are also cases when a semi-cooperative triplet scores a 5 or 6 in the second level classification. In this situation, there is at least one genotype which gains no net benefit from being in the triplet relative to being isolated but which gains an advantage relative to being in a pair with either other member of the triplet. Interestingly, we find that there are a few antagonistic triplets, which score a 6 on the second level classification. This situation implies that each genotype in such a triplet does worse in combination with both of the other two, however if any two genotypes are present, they both gain an advantage by adding the third. These genotypes have the highest growth rates alone, but if they cannot be isolated completely they are better off in the triplet. These are just some examples of the types of evolutionary trade-offs present in this chemical system.

Understanding the evolution of cooperation in chemical systems will be essential to understanding the complexification of such systems as they make the transition to biological ones. In the current study, we tested the effect of the distribution of rate constants for ribozyme assembly. As can be seen in Figure 4, this distribution has a substantial effect on how fitness is partitioned between individual and group benefits. These rate constants in turn are determined by the chemical moieties in the base-pairing surface of a single nucleotide pair embedded in the middle of the IGS–tag triplet–triplet interactions. The steric and electronic configurations of this base-pairing surface in turn is determined by only a few atomic rearrangements, which in some cases involve as few as three atoms (compare a G-C pair with a G-U pair, for example). Thus here we are able to draw a line of causality from the atomic level to the group population level dynamics of complex macromolecules with molecular masses greater than 50,000 daltons. The dynamical behavior of ribozyme self-assembly then may be another manifestation of (or even determinant of) the chemical etiology as discussed by Eschenmoser [22].

One of the most salient features of the biosphere is its hierarchical organization [7,23,24,25,26]. The emergence of biological hierarchies can be viewed as a type of dynamic computation, where component parts are both partially constrained by and partially responsible for the higher-level organization that emerges [24]. Notably, one of the key features of the population level organization observed in the *Azoarcus* RNA system was due to the heterogeneous distribution of rate constants. In other aspects of biology, heterogeneous distributions of connections have been shown to enhance the information-processing capabilities of biochemical networks [27,28]. It is possible that the distribution of base-pairing energetics in nucleobases provides RNA systems with enhanced computational and informational abilities relative to other plausible prebiotic chemistries. That enhancement would have contributed to the ability of RNA-based systems to generate higher levels of hierarchical organization.

## 4. Materials and Methods

### 4.1. Kinetic Simulations

To explore the drivers of cooperation in this system, we developed a dynamic model of *Azoracus* ribozyme self-assembly. In contrast to previous models of this system, we considered a closed system with a fixed total number of **WXY** and **Z** fragments. We model the self-assembly process as a spontaneous reaction which can be catalyzed by assembled ribozymes. The spontaneous assembly propensity for a given genotype is determined by the total abundance of **WXY** fragments of that genotype and the number of **Z** fragments and the reaction rate constants $k_{s}$, such that the rate is proportional to $k_{s}\left[WXY_{i}\right]\left[Z\right]$. The assembly of ribozymes can be catalyzed by other ribozymes. The degree of catalysis is determined by the IGS of the completed ribozyme and the tag of the ribozyme to be assembled. Catalysis increases the spontaneous propensity of assembly by a factor of $k_{ij}\left[X_{i}\right]$, where $k_{ij}$ is the degree to which a ribozyme with IGS *i* catalyzes the assembly of ribozymes with tag *j*, and $\left[X_{i}\right]$, is the number of ribozymes with IGS *i*. Thus the total assembly propensity of a ribozyme with tag *j* is proportional to

(1) $$\begin{array}{c} A_{j}\propto k_{s}\left[WXY_{j}\right]\left[Z\right]\left(1+\Sigma_{i}k_{ij}\left[X_{i}\right]\right). \end{array}$$

For the results presented here we have set the spontaneous rate constant to a small value ($k_{s}=10^{-8}$), and we have approximated the values using the self-assembly autocatalysis rate constants provided in [6]. This model was implemented in a kinetic Monte Carlo algorithm [29].

### 4.2. Fitting Growth Constants

Understanding cooperation in this system requires comparing the growth rates of genotypes when they are isolated, and when they are coexisting with other genotypes. In order to make this comparison simulations were performed with genotypes individually, in pairs, and in triplet groups. For the individual simulations, the system was initialized with one completed ribozyme of the given genotype, 5000 **WXY** fragments of that genotype and 15,000 **Z** fragments. For simulations with pairs of genotypes, the system was initialized with two complete ribozymes (one of each genotype), 10,000 **WXY** fragments (5000 of each genotype) and 15,000 **Z** fragments. Simulations with triplets were performed in a similar manner, with three completed ribozymes (one of each genotype), 15,000 **WXY** fragments (5000 of each genotype) and 15,000 **Z** fragments. For each simulation time series data for the abundance of each ribozyme genotype was recorded. To compare the growth rate of a given genotype under different conditions (e.g isolated, in triplet, or pair), an exponential curve of the form,

(2) $$\begin{array}{c} X_{i}^{C}\left(t\right)=X_{0}\exp\left(R_{i}^{C}t\right), \end{array}$$

was fit to the beginning of simulated time series data, where $X_{i}^{C}$ is the abundance of the completed ribozyme of genotype *i*, in condition *C*, and $X_{0}$ is the fitted growth rate for genotype *i*, in condition *C*. The possible conditions for a given ribozyme, are alone which we denote C=i for ribozyme *i*, in pairs which we denote C=ij for the combination of ribozymes and *i* and *j*, or in triplets which we denote C=ijk.

### 4.3. Randomization Experiments

In order to understand what drives the observed distribution of cooperation, we compared the distribution of cooperative ribozymes derived from the empirical catalytic rate constants to the distribution of cooperative ribozymes derived from other catalytic rate constants (e.g., different values for Table 1). Several different distributions of reaction rate constants were chosen to understand which features of the catalytic rate constants caused the observed distribution of rate constants. As a control, rate constants were drawn from a random uniform distribution with a range equal to the range of the empirical constants (0.0001, 0.0415). We refer to these as flat rate constants. Since the self-assembly process in the *Azoracus* ribozyme depends on the nucleobases in the IGS and the tag, the rate of catalytic reaction rate constants for any Watson–Crick pair of IGS and tag nucleobases are much higher. Therefore the observed distribution of catalysis may be due to the spacing between the highest rate constants and the lowest. To capture these features we use three different synthetic distributions, to compare to the real distribution (Table 1). The first distribution, which we refer to as the Watson–Crick (WC) distribution, assigns the Watson–Crick IGS–tag pairs to have a uniform catalytic rate constant (0.0400), which is two orders of magnitude higher than all the other rate constants(0.0004). We also used another distribution in which we again assign Watson–Crick IGS–tag pairs to have a high uniform rate constant (0.0400), which is one order of magnitude above the rate constant assigned to “wobble pair” IGS, tag pairs (0.0040), and all other rate constants were set to be one order of magnitude lower than the wobble pair constants (0.0004). We refer to this distributions as the Watson–Crick–Wobble (WCW3) distribution. The final distribution is one where rate constants are uniformly distributed on a logarithmic scale, which we refer to as the log random or heterogeneous rate constants.

## 5. Conclusions

The evolution of complexity is typically explained by understanding how individuals form cooperative groups. In the chemical events surrounding the origins of life, it appears that explaining the converse situation—how individuals arise from cooperative groups—will be an important task. The advent of cells or some type of semi-permanent encapsulation mechanism allowed for the physical linkage of genotypes with their own phenotypes, as well as energetic benefits [12,30,31,32]. This ”invention” of a barrier, such as proto-cells, likely led to an evolutionary advantage to individuals. However, reliably passing on the key molecular machinery to new containers likely would have required specialized chemical processes. Specialization to that degree requires a division of labor amongst prebiotic molecules, and it is well-known in evolutionary theory that cooperation is a prerequisite of division of labor [25]. For these reasons we suggest that in a prebiotic chemical soup, the rules of cooperation such as those we investigated here, would have been the primary drivers of evolutionary change.

## Figures and Tables

**Table 1:** Autocatalytic rate constants for *Azoarcus* RNA covalent self assembly.

WXY Genotype	Self-Assembly Rates ($k_{a}$); min^−1^	Nucleotide Pair Type
C•G	0.0415	
A•U	0.0319	strong (Watson–Crick)
U•A	0.0197	
G•C	0.0125	
G•U	0.0091	
A•C	0.0069	intermediate (wobble)
U•G	0.0049	
U•C	0.0038	
U•U	0.0022	
C•A	0.0020	
C•C	0.0016	
G•G	0.0006	weak
G•A	0.0005	
A•A	0.0004	
C•U	0.0004	
A•G	0.0001	

## Abbreviations

The following abbreviations are used in this manuscript:

RNA	Ribonucleic acid
IGS	Internal guide sequence
RPS	Rock-paper-scissors
G	guanosine
A	adenosine
C	cytidine
U	uridine
WC	Watson–Crick
WCW3	Watson–Crick 3 Wobble Pairs
